# Inappropriate usage of selected antimicrobials: Comparative residue proportions in rural and urban beef in Uganda

**DOI:** 10.1371/journal.pone.0209006

**Published:** 2019-01-10

**Authors:** Yake Basulira, Susan A. Olet, Paul Erasmus Alele

**Affiliations:** 1 Department of Pharmacology and Therapeutics, Faculty of Medicine, Mbarara University of Science and Technology, Mbarara, Uganda; 2 Department of Biostatistics, Aurora Research Institute, Milwaukee, Wisconsin, United States of America; The University of Sydney, AUSTRALIA

## Abstract

**Introduction:**

In most developing countries like Uganda, antimicrobials including β-lactams and tetracyclines are used indiscriminately in livestock. When livestock get sick and treatment is necessary, some producers and veterinarians use these drugs with minimal controls to prevent residues from occurring in the beef sent to markets. This study was done to determine the presence of drug residues above acceptable limits of two commonly used antimicrobials in Uganda’s rural and urban beef.

**Methods:**

A cross-sectional study was conducted of 134 cattle carcasses from eight different slaughter slabs over twelve weeks. This study entailed 81 samples of rural and 53 samples of urban origin. To enable detailed analysis these samples were categorized according to age (maturity), breed, and sex. For each of the 134 carcasses, three samples of liver, kidney and muscle were taken and homogeneously mixed into one sample, which was tested for β-lactam and tetracycline drug residues.

**Results:**

The results were statistically significant for β-lactam levels (χ^2^ = 22.10, df = 10, p = 0.0146) with average concentration (μg/kg) of 2.93:29.3 (rural: urban), though not for tetracycline levels (χ^2^ = 3.594, df = 10, P = 0.9638) with average concentration (μg/kg) of 5.028:12.83 (rural: urban). Age (maturity) had significant effect at all values of antibiotic level (F_(1, 68)_ = 5.06, p = 0.0278). Age effect was extremely significant (F_(1, 68)_ = 15.51, p = 0.0002).

**Conclusion:**

A significant difference existed in drug residue proportions of β-lactam and tetracycline antimicrobials among Uganda’s rural and urban beef. A significant difference also occured in drug residue proportions of these two commonly used antimicrobials related to age (maturity), but neither breed, nor sex, of Uganda’s rural and urban beef.

## Introduction

The irrational use of antimicrobials is now recognized as a major global health concern especially in developing countries where regulation on the use of drugs is inadequate [1]. One of the irrational ways of using antimicrobials is to routinely administer these drugs to livestock, often by giving the same antimicrobials as those used by humans [2]. Drugs are generally used on farm animals for therapeutic and prophylactic purposes and include a large number of different types of compounds that can be administered in the feed or in the drinking water [2]. Antimicrobials have been used in livestock farming for several decades in combating bacterial infections, but inappropriate usage can lead to the occurrence of residues in foods of animal origin particularly meat, milk, and eggs. In most developing countries, antimicrobials like tetracycline, erythromycin, streptomycin, and penicillin are used in animals indiscriminately for the prevention and treatment of bacterial infection [3]. Growing concern among consumers about antibiotic and other drug or chemical residues in meat, has increased emphasis from the regulatory agencies in Uganda to monitor and prevent drug residues in foods of animal origin through training programs for veterinary service providers and licensing of personnel allowed to handle and administer veterinary drugs [4,5]. These changes have come about because of increased sensitivity of the tests available [6] and the resultant enforcement actions and penalties for violations by regulatory agencies. Farm animals treated with antimicrobials are required to be held for a specific withdrawal period until all residues are depleted to a safe level before the animal tissue can be used as food for human consumption [7].

Drug residues, which are drug molecules present in the tissues of a treated animal, are most commonly the result of failure of elimination of the drug from the edible portions of the animal because insufficient time was allowed between the last treatment and slaughter of the animal [8]. The ensuing risks of pharmaceutical residues reaching edible products and the potential health hazards associated with their consumption have become a public safety issue [9,10]. It is now recognized that these drugs may exert other effects other than those for which they are administered, including allergic reactions and selection of resistant bacteria that could be transferred to humans through the food chain, changes in the resistance patterns of bacteria exposed to antibiotic, and direct toxic effects [2,11,12]. This is important, given that approximately 5–10% of the population worldwide is hypersensitive to penicillin or other antimicrobials and suffers allergic reactions (skin rashes, hives, asthma, anaphylactic shock) at concentrations as low as 1 ppb penicillin [8]. Residues of antibacterial drugs have been reported in slaughtered beef cattle, veal calves, and pork [13–15], affecting the indigenous human intestinal microflora which constitutes an essential component of human physiology [16–18]. These microflora act as a barrier against the colonization of the gastrointestinal tract by pathogenic bacteria, and have an important role in food digestion. Standard regulatory protocols have been established in many countries to avoid the slaughtering of animals harboring residues [19].

Tetracyclines and penicillins are two antibiotics whose residues have been identified in beef over time [16,20–22]. Tetracyclines are broad-spectrum antimicrobials, which are widely used in animal husbandry for both prevention and treatment of diseases and for growth promotion in food-producing animals [22]. Due to enterohepatic circulation, a small amount of the administered dosage of tetracyclines may persist in the body for a long time after administration. Tetracyclines such as oxytetracycline are available as over-the-counter drugs, are inexpensive [23] and were the commonest antimicrobial available in homesteads of rural cattle-keepers in a recent study [23] in Tanzania. Additionally, in a recent study in Burkina Faso, tetracyclines (oxytetracycline) were the most frequently sold veterinary antibiotic to animal breeders [13]. In Uganda, however, data on the frequency of use and indications for use in veterinary care are lacking. Penicillins are another group of antimicrobials that have been found to be commonly used among cattle owners in Tanzania [23]. Penicillins, a type of β-lactam antibiotic, are sometimes combined with streptomycin (Penstrep) and are also available over-the-counter [23]. Like the tetracyclines, penicillins are inexpensive, and so are commonly used for veterinary care of cattle by rural cattlekeepers [23]. In Burkina Faso, penicillins (penicillin G and Penstrep) were the second most commonly sold antibiotics in veterinary pharmacies and clinics [13]. Tetracycline and penicillin residues found in beef above tolerable levels can occur because of the permissive and uninformed use of these drugs by segments of the animal industry; the failure to adhere to the withdrawal regulations; the extra-label use of drugs for which there may be no stated withdrawal period; and, the lack of original owner identification for animals offered for slaughter [24].

Recent estimates of the total cattle livestock number in Uganda showed an increase from 14 million in 2015 to 14.4 million in 2016 [25]. Indigenous cattle breeds are predominant (93%), the rest being exotic breeds. Of the total cattle population, an estimated 90% is kept under pastoral and mixed smallholder farming systems, with commercial beef ranching accounting for less than 10% of the total cattle number [25]. Recent statistics available from the animal industry in Uganda show that beef production increased from 209000 metric tonnes in 2015 to 214000 metric tonnes in 2016 [25]. Several regulatory bodies exist in Uganda, including the Ministry of Agriculture, Animal Industry and Fisheries; the Uganda National Bureau of Standards; the National Environment Management Authority; and the Uganda Beef Producers Association [5]. Uganda has had a policy on veterinary drugs for nearly two decades now [4]. Besides focusing on veterinary drug quantification, manufacturing, procurement, distribution and usage, the policy specifically mentions the monitoring of drug residues in foods of animal origin, and the correct and safe use of veterinary drugs, as major areas of focus [4]. One of the key guiding principles of this policy is that the proper use of veterinary drugs shall be regularly monitored to reduce risks associated with public health and development of resistance to drugs and chemicals [4]. Despite the available policy, its implementation has not been adequate owing to lack of a legal framework, lack of infrastructure such as laboratories, and lack of personnel to periodically enforce the policy [4,5,25].

Most cattle farmers in Uganda who keep animals for meat do so using smallholder cattle production systems where cattle are mostly communally grazed [26]. Cattle and other animals (goats, sheep and pigs) are reared alongside crop-growing in many parts of both rural and urban Uganda [26]. In Ntungamo, in southwestern Uganda, cattle are mainly communally grazed and also paddocked [26]. Poor production practices involving extra-label drug use are commonly acknowledged to be present in veterinary medicine globally [27,28]. Extra-label usage can be divided into two separate areas: use of drugs which have not been approved for the species in question (e.g. use of poultry antimicrobials to treat cows), and use of approved drugs at levels which differ from the recommended dosage. Failure to follow recommended withdrawal times is often implicated in residue problems [28].

Our research goal, therefore, was to determine whether there are significant drug residue proportions and differences in drug residue proportions of tetracyclines and β-lactams, two commonly used antimicrobials, in rural and urban beef in Uganda. We defined significant drug residues as those occurring above the acceptable limit of 30 μg/kg body weight [29]. We hypothesized that significant drug residue proportions exist in rural and urban beef and that differences in drug residue proportions of these commonly used antimicrobials in Uganda’s rural and urban beef would exist based on sex, age, and breed of the cattle. Specifically, we wanted to determine and compare drug residue proportions of the selected commonly used antimicrobials in rural and urban beef in relation to sex, age and breed of cattle.

## Materials and methods

### Study area

We conducted research in Ntungamo district as a representative rural district and in Kampala district as a representative urban district. We purposively chose to study Ntungamo district as a representative rural district because it is located in southwestern Uganda where over 50% of the cattle population of Uganda is found [25,30]. The average number of cattle per household in this region is 2.11 compared to eastern and northern Uganda at 0.67, and the national average at 1.37 cattle per household [4]. Kampala district was chosen because it lies along the “cattle corridor” that extends from southwestern to northeastern Uganda where most beef production occurs [5]. Sample storage and pre-treatment were done at the district laboratories in Ntungamo, at Wandegeya Central Government Laboratories, Kampala, and at Chemiphar (U) Ltd / Eurofins (U) Ltd Kampala. Laboratory studies were carried out at the Wandegeya Central Government Laboratories, Kampala, and at Chemiphar (U) Ltd / Eurofins (U) Ltd, for the drug residue analysis.

### Study population

The rural study population included cattle brought for slaughter in the 4 slaughter places of Ntungamo (Kitwe, Rubare, Rwashameire, and Ntungamo); variable cattle numbers were chosen from each slaughter place. The urban study population included cattle brought for slaughter in the 4 abattoirs of Kampala (Kalerwe, Wankulukuku, Kampala Meat Packers and Kampala City Abattoir). Beef samples were chosen from each of the abattoirs. In Uganda, the slaughter industry generally consists of domestic family slaughter; slaughter at village markets for sale, where cattle, goats and other animals are slaughtered on slabs or sometimes on the ground; town slaughter slabs–the majority of towns (approximately 80%)–have slaughter slabs for supplying meat to town residents and restaurants; and abattoirs [30]. The eight slaughter facilities from which we obtained beef samples represent the places where most beef consumed in Uganda are obtained.

### Sample size and procedure

**Fig 1:**
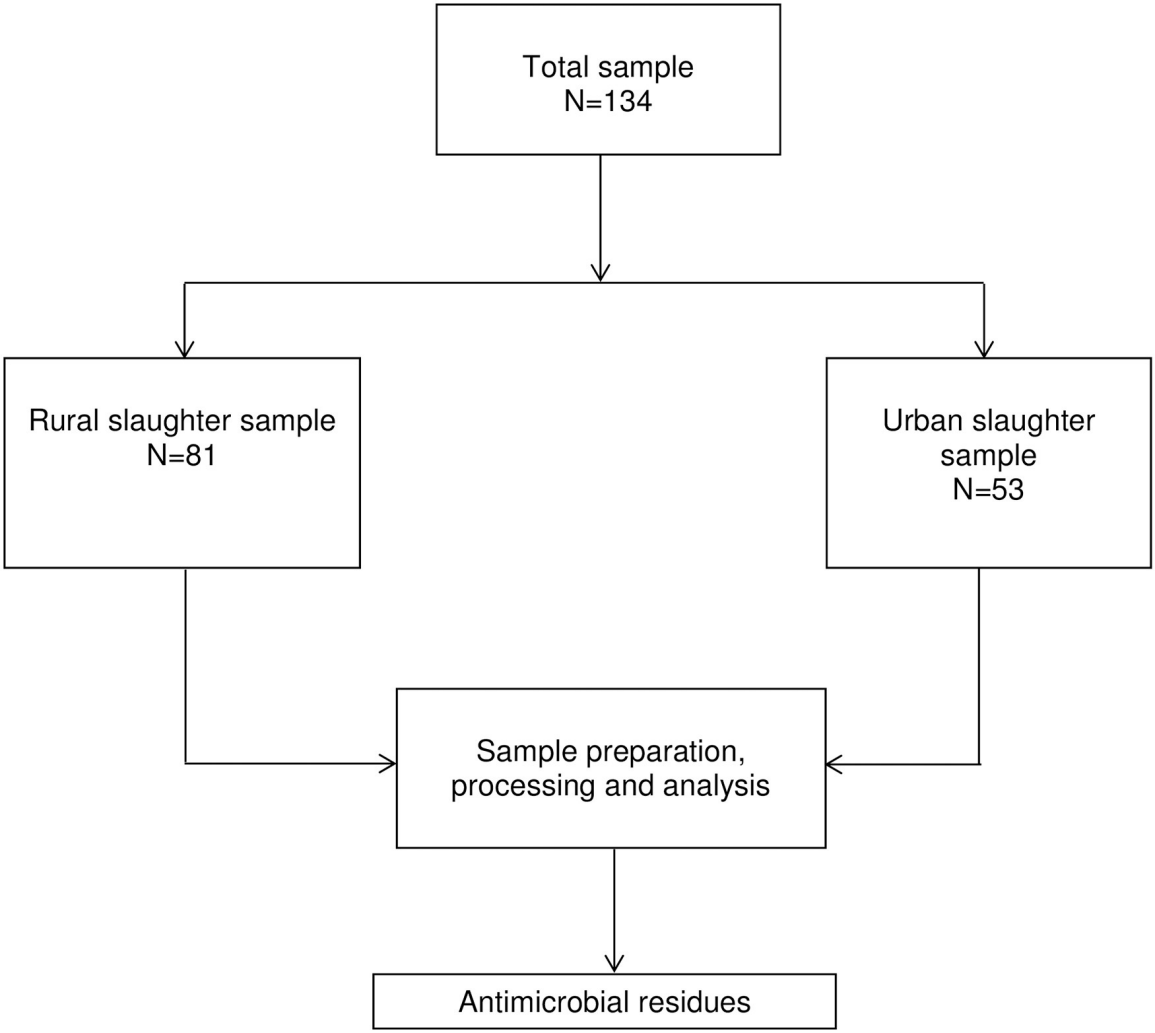
Flow chart of tissue sampling.

We estimated that at least fifty carcass samples from each location (rural and urban) would detect significant odds ratios based on 80% power and 95% confidence. Using the Kish formula [31], sample size, N = Z^2^p(1 –p)/d^2^, where p = prevalence; Z = score of confidence interval at 95% (CI = 1.96); d = tolerable error (absolute precision) of 5%. Using a prevalence of 31% for antimicrobial presence in beef screened in a recent published study [13], sample size was calculated as 148. We obtained 134 samples representing 90% of the calculated sample size (81 from rural Ntungamo and 53 from urban Kampala; Fig 1). Each sample was analyzed for two antibiotic groups, the beta-lactam and tetracycline, attaining 268 sample readings, 48 standard readings and 16 controls (332 readings).

Both rural and urban carcasses from the slaughter places were sampled using a purposeful sampling method. Sampling was done on different days for the urban and the rural slaughterhouses and occurred over a period of 12 weeks. Dealers sell the animals supplied to the slaughterhouses from diverse locations within the rural locale, and the urban areas. We obtained information on the farm of origin for each carcass based on records available from the slaughterhouses, and from animal transfer permits allowing the transport of animals to the slaughterhouses. Cattle brought in at the slaughterhouses are recorded, and kept at the facility grounds for a day or two prior to slaughter. During this time, we selected those cattle that met our inclusion criteria (below) for each day at the facility. We, however, did not physically track the farms from which each animal carcass originated. Carcass samples were assigned numbers. Numbers were given to only those carcasses that represented sampled cattle of interest. However, samples were arranged into four categories (young, adult, female, and male) and two groups (local and crosses/exotic breed) from each slaughter place. This was done to consider the breeds separately since urban farmers could have more of the cross/exotic than the local breed. Samples included liver, kidney and muscle (thigh and/or neck) of cattle carcasses. Carcasses were selected for inclusion in the study if the slaughtered cattle were older than six months, the beef did not show bruising or sites of recent injection, and the cattle were brought in to the abattoir from a 30 km radius within Kampala city (urban samples) or from a rural farm within Ntungamo (rural samples). Carcasses were consecutively sampled from the slaughtered animals if they met these criteria. Samples that were excluded from the study included beef of young calves less than six months of age; beef of home and bush slaughters (slaughters from non-gazetted places); and, beef from bruised carcasses or carcasses still showing sites of antibiotic injection.

### Study design and tissue processing

This study was an analytical observational cross-sectional study that was conducted with purposeful sampling of cattle carcasses over a period of twelve weeks. We obtained samples of beef carcasses from four rural abattoirs and four city abattoirs (Fig 1) to estimate the concentration of beta-lactam and tetracycline in beef originating from rural and urban locations. We did not administer any treatments ourselves. However, because our interest was to determine the concentration of the two commonly used antimicrobials (beta-lactams and tetracycline) in beef originating from rural and urban places, the experimental units were the four rural and four urban locations represented by the abattoirs from which the carcasses were sampled. Our sampling units were the individual carcasses from the abattoirs (Fig 1). Sampled carcasses were numbered serially from 1 to 81 (for rural cattle) and 82 to 134 (for urban cattle). Cattle carcasses were further identified according to sex, age and breed of cattle slaughtered. Samples (about 200 g) of the liver, kidney, and muscle each from the same carcass were collected, split into about 1 g pieces each, ground, precipitated, and filtered, and the supernatant redissolved.

Tissue processing and dilution was done as follows: 1 g of liver, or kidney or muscle was finely chopped and added to 9 ml of 0.1M phosphate buffer (pH 6.5). The tissue was homogenized for 30 seconds, followed by vortexing for 1 minute, and then centrifuged at 4000 rpm for 20 minutes at 4°C. The supernatant layer was decanted off, filtered using an acrodisc syringe filter; if the sample was particularly fatty, glass wool was used to filter sample per manufacturer recommendations. The filtered sample was diluted in a 2:1 ratio in diluted wash buffer and applied to the microtitre plate. Filtered samples for the muscle, liver, and kidney from the same carcass were homogeneously mixed into one sample, before dilution.

### Materials, stability and preparation of reagents

The procedures described were adapted from Randox [32]. The microtitre plate was obtained ready for use. Per manufacturer recommendations, if only part of the plate was being used, the remaining strips were returned to the foil grip bag with desiccant, and resealed, ensuring that excess air was expelled. The microtitre plate is stable up to expiry when stored at +2 to 8°C. The concentrated diluent/wash buffer was diluted by adding the contents of one vial of diluent/wash buffer, to 970 ml of double deionised water. The concentrated diluent/wash buffer is stable for 30 days at +2 to +8°C. The conjugate is supplied as a freeze-dried concentrate. Once diluted the conjugate should be used immediately, and should be protected from light. The One-Shot Substrate is supplied ready to use, and does not contain any harmful organic solvents; it is stable up to expiry when stored at +2 to 8°C. Similarly, the Stop solution is supplied ready to use and is stable up to expiry when stored at +2 to 8°C. All products were handled with care using latex gloves, and a respiratory protection device worn. Stock solutions of the pharmaceuticals to be studied were supplied in the highest available purity, and prepared per the catalogs. Samples were transported in sample bottles, iceboxes, and ice packs.

### Analytical procedure

The analytical procedure used was adapted from Randox [32]. All reagents attained room temperature (+19 to +25°C) per manufacturer recommendations, prior to use. Microtitre plates were sealed with an adhesive plate sealer or cling film to prevent evaporation. Standard (50 μl), sample (50 μl), quality control (50 μl) and conjugate (75 μl) were pipetted into the appropriate wells of the microtitre plate. The microtitre plate was then gently tapped from side to side for a few seconds and covered with adhesive film before incubating for 1 hour at room temperature (+19 to +25°C) in the dark. Thereafter, the plate was inverted and the liquid tapped out; the plate was then washed 6 times using diluted diluents /wash buffer (ensuring that every well was filled), over a 10–15 minute period. After the final wash, the liquid was discarded and the plate tapped onto tissue paper until completely dry. Immediately after washing, 125 μl of the One-Shot Substrate solution was pipetted into each well using a multichannel pipette. The microtitre plate was gently tapped from side to side and incubated for 20 ± 2 minutes at room temperature (+19 to +25°C) in the dark. The color reaction was stopped by the addition of 100 μl of Stop solution per well. A color change from blue to yellow was evident. Optical density was measured at 450 nm within 10 minutes of stopping the color reaction.

### Data analysis

The data were analysed using SAS version 9.4, and tests were performed at a 5% level of significance. The procedures used are described below. Continuous characteristics are presented as median [lower quartile (Q1)–third quartiles (Q3)] while categorical characteristics as counts and proportions. Bar graphs display a comparison of beta-lactam and tetracycline antimicrobials levels separately by age of the cattle carcasses. Normality was assessed using Shapiro-Wilk’s and Kolmogorov-Smirnov tests where a p-value greater than 5% implied that inference based on a parametric test was valid. Based on World Health Organization and Food and Agriculture Organization standards, we categorized the antibiotic residues in the beef samples as negative (<10 μg/kg body weight), within normal/acceptable limits (10–30 μg/kg body weight), and above acceptable levels (>30 μg/kg body weight) [29]. To assess the difference between negative, acceptable or above limit categories the chi-square test of association was performed for categorical variables while the Wilcoxon rank-sum test was used for continuous variables. We conducted univariate analysis to investigate associations and comparisons between variables in response to the two outcomes, tetracycline and beta-lactam levels. The variables of interest included age, breed, sex, and place of origin. Univariate analyses were conducted using Chi-square and Wilcoxon rank sum tests as appropriate. As an initial step, we considered the response as categorical-ordinal. A multivariate model was then fitted adjusting for covariates of interest. The multivariate model that was fitted was the Proportional Odds Model in the generalized linear model (GLM) family. Both tetracycline and beta-lactam were categorized using similar cut-offs to obtain an ordinal variable(s) 0 = Negative (**0** ≤ residual level ≤ **10**, 1 = Acceptable/Normal (1**0** < residual level ≤ **30**, 2 = Above Acceptable (residual level > **30**).

Further analyses entailed obtaining odds ratios and their associated 95% confidence limits (CL) to quantify the direction and magnitude of the beef sample characteristics on fitting a proportional odds model. The procedure Mixed (Proc Mixed) was fitted to account for the association among cattle samples’ origin. The origin of cattle was modeled as a random effect, while the antibiotic concentration was modeled as a fixed effect. All tests were performed at 5% level of significance.

### Ethical considerations

The research plan was reviewed and approved by the Faculty of Medicine Research Committee (DMS 6–2012) and the Research Ethics Committee of Mbarara University of Science and Technology (No. 10/08-12).

## Results

Of 134 beef samples of cattle carcasses analyzed, 60.4% (81) were of rural origin, while 39.6% (53) of urban origin. The majority of the samples were of rural origin (60.4%), females (56%), adult (62.7%), and local breed (65.7%). Among the beef samples analyzed, a higher proportion (88.1%) were negative (<10 μg/kg body weight) while 6.7% were above acceptable levels (>30 μg/kg body weight) for tetracycline. On the contrary, a higher proportion (61.9%) of beef samples analyzed for beta-lactam were above acceptable limits and a lower proportion (15.7%) of the samples analyzed were negative (Table 1).

**Table 1:** Characteristics of cattle carcass samples.

Characteristics	Frequency (n = 134)	Percent (%)
**Age**		
Adult	84	62.7
Young	50	37.3
**Sex**		
Female	75	56
Male	59	44
**Place**		
Rural	81	60.4
Urban	53	39.6
**Breed**		
Local	88	65.7
Cross	46	34.3
**Antimicrobial Concentrations**		
**Tetracycline**		
Negative	118	88.1
Normal	7	5.2
Above acceptable	9	6.7
**Beta-lactam**		
Negative	83	15.7
Normal	30	22.4
Above acceptable	21	61.9

The median β-lactam concentration level in urban beef samples was at least tenfold that of rural beef samples (26.0 μg[Q1: 16.8 –Q3:37.5] vs 2.51 μg[Q1: 2.07 –Q3:3.02]; p <0.0001; Table 2). Conversely, the median tetracycline concentration level in rural beef samples was higher than in urban beef samples (4 μg [Q1:2.5 –Q3:6.1] vs 2.5 μg [Q1:0.9 –Q3:5.2]; p = 0.0403). The median β-lactam concentration level was higher among the adult carcass beef samples compared to the young carcass beef samples with a statistically significant difference (p = 0.0048; Table 2). The concentration levels for both the beta-lactam and tetracycline antimicrobials, were associated with place of origin (rural: versus urban; p < 0.05; Table 2). Age also statistically predicted significant differences in beta-lactam levels but not in tetracycline levels (Tables 2 and 3). The contribution of breed to predicting the beta-lactam level was marginally significant (p < 0.081).

**Table 2:** Antimicrobial concentration levels in cattle carcass samples.

Characteristics	Beta-lactam (μg/kg)		
	n	Median (Q1—Q3)	P value
**Age**			0.0048*
Adult	84	7.6(2.4–28.0)	
Young	50	**2.8(2.3–7.6)**	
**Sex**			0.6982
Female	75	3.4(2.3–17.8)	
Male	59	3.1(2.4–26.4)	
**Breed**			0.0805
Local	88	4.4(2.4–25.4)	
Cross	46	3.0(2.2–11.1)	
**Place**			< .0001*
Rural	81	2.51(2.1–3.0)	
Urban	53	**26.0(16.8–37.5)**	
	**Tetracycline (μg/kg)**		
**Age**			0.9523
Adult	84	3.9(1.9–6.1)	
Young	50	3.5(2.4–6.1)	
**Sex**			0.3621
Female	75	3.7(2.1–5.8)	
Male	59	4.0 (2–6.9)	
**Breed**			0.8534
Local	88	3.9(2.3–6.1)	
Cross	46	3.2(2–6.1)	
**Place**			0.0403*
Rural	81	4(2.5–6.1)	
Urban	53	**2.5(0.9–5.2)**	

Q1-Lower Quartile, Q3-Upper Quartile;

*p<0.05 comparing the differences in antimicrobial residue levels between adult and young cattle and cattle from rural and urban places of origin.

**Table 3:** Association of antimicrobial residues and cattle carcass characteristics.

Characteristics	Overall	Negative	Acceptable/Normal	Above Acceptable	P-value
**β-lactam**					
**Sex**					
Female	75(56.0%)	47(56.6%)	19(63.3%)	9(42.9%)	0.343
Male	59(44.0%)	36(43.4%)	11(36.7%)	12(57.1%)	
**Age**					
Young	50(37.3%)	41(49.4%)	6(20.0%)	3(14.3%)	0.0009*
Adult	84(62.7%)	42(50.6%)	24(80.0%)	18(85.7%)	
**Breed**					0.1188
Local	88(65.7%)	49(59.0%)	23(76.7%)	16(76.2%)	
Cross	46(34.3%)	34(41.0%)	7(23.3%)	5(23.8%)	
**Place**					na
Rural	81(60.4%)	79(95.2%)	2(6.7%)	0(0.0%)	
Urban	53(39.6%)	4(4.8%)	28(93.3%)	21(100.0%)	
**Tetracycline**					
**Sex**					0.513
Female	75(56.0%)	68(57.6%)	3(42.9%)	4(44.4%)	
Male	59(44.0%)	50(42.4%)	4(57.1%)	5(55.6%)	
**Age**					0.2601
Young	50(37.3%)	46(39.0%)	3(42.9%)	1(11.1%)	
Adult	84(62.7%)	72(61.0%)	4(57.1%)	8(88.9%)	
**Breed**					0.7678
Local	88(65.7%)	76(64.4%)	5(71.4%)	7(77.8%)	
Cross	46(34.3%)	42(35.6%)	2(28.6%)	2(22.2%)	
**Place**					
Rural	81(60.4%)	77(65.3%)	4(57.1%)	0(0.0%)	na
Urban	53(39.6%)	41(34.7%)	3(42.9%)	9(100.0%)	

There was a statistically significant difference (*p<0.05) comparing the β-lactam residues among young and adult cattle carcasses.

**Fig 2:**
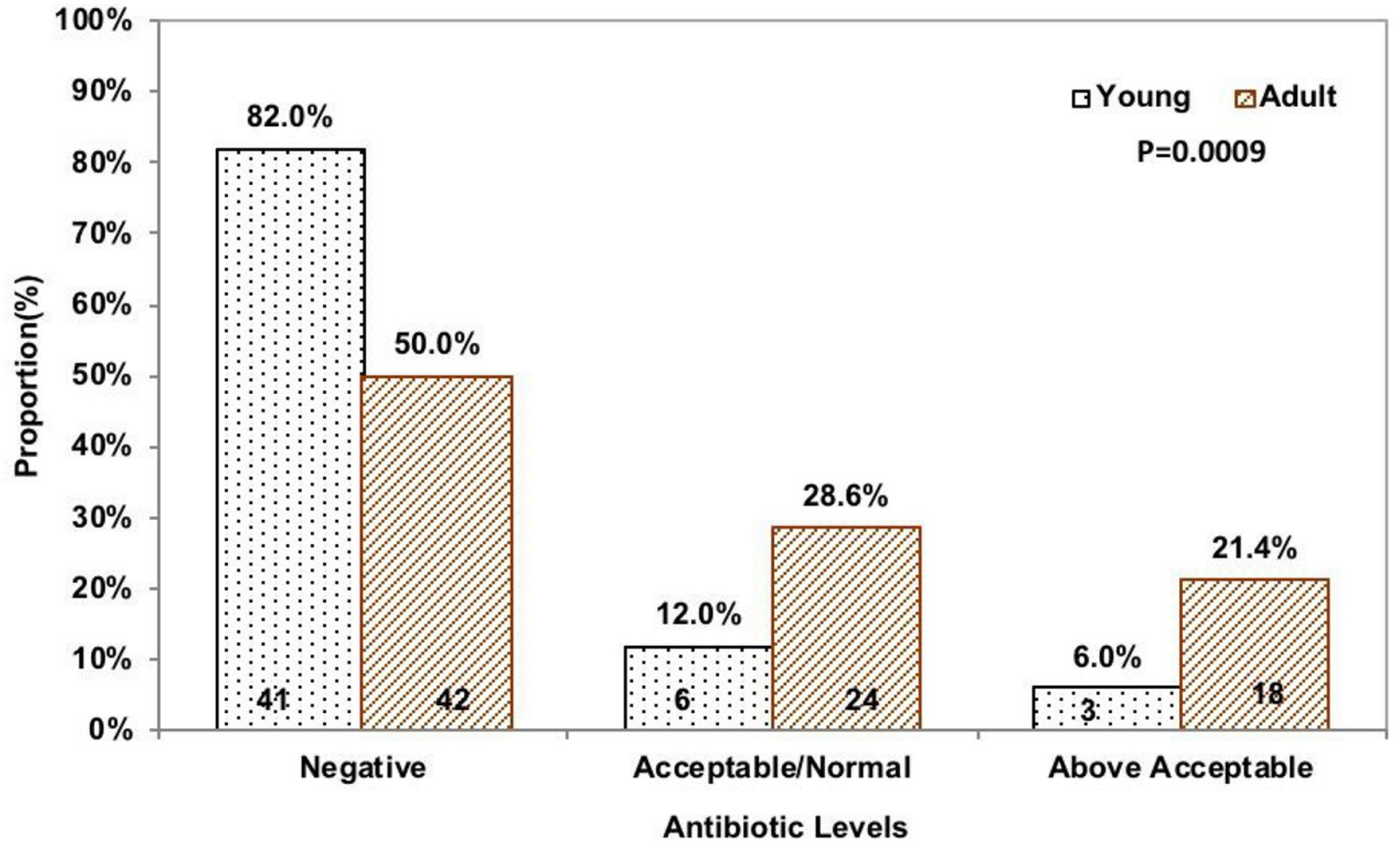
Beta-lactam levels by age (beef carcass counts are at the bottom of bars).

**Fig 3:**
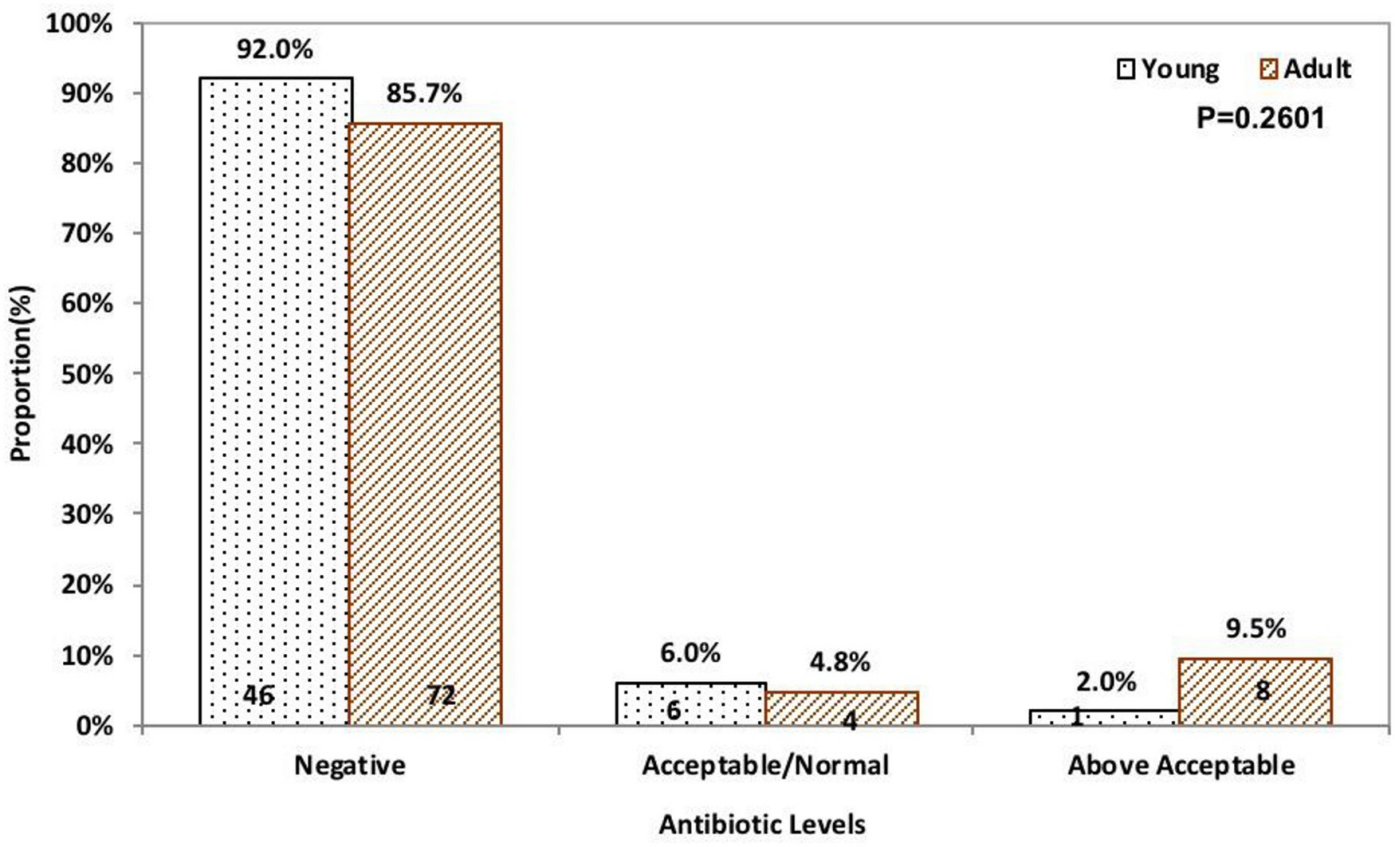
Tetracycline levels by age (beef carcass counts are at the bottom of bars).

Beta-lactam and tetracycline antimicrobial residues were categorized into negative, normal, and above acceptable limits. Of 21 beef samples that had above acceptable beta-lactam residues, 85.7% (18/21) were adult cattle carcass samples. Fig 2 shows visually that the proportion of beef carcass samples from young cattle with beta-lactam residues above acceptable levels were lower compared to the beef samples from adult cattle and the difference was statistically significant (p = 0.0009). Similarly, of 9 beef samples that had above acceptable tetracycline residues, 88.9% (8/9) were adult cattle carcass samples. Fig 3 shows visually that the proportion of beef carcass samples from young cattle with tetracycline residues above acceptable limits were lower compared to the beef samples from adult cattle although the difference was not statistically significant (p = 0.2601).

Analysis of place of origin and cattle carcass characteristics showed age and breed were associated with place of origin (p <0.05). A higher proportion of beef samples from young cattle carcasses (48.1%) and crossbreed (43.2%) were of rural origin compared to the beef samples from adult cattle carcasses (20.8%). The difference in proportion was significantly different; p values < 0.05 (Table 4).

**Table 4:** Associations between place of origin and cattle carcass characteristics.

Characteristics	Place of Origin			Odds Ratio	95% CL	P- value
	Overall n = 134(%)	Rural n = 81(%)	Urban n = 53(%)			
**Sex**	59(44.0%)	38(46.9%)	21(39.6%)	1.35	0.67–2.72	0.4058
**Age (Young)**	50(37.3%)	39(48.1%)	11(20.8%)	3.54	1.60–7.84	0.0013*
**Breed**	46(34.3%)	35(43.2%)	11(20.8%)	2.91	1.31–6.43	0.0074*

The asterisk (*) denotes a statistically significant difference comparing the rural and urban cattle samples with respect to age and breed of the cattle carcasses.

Separate proportional odds models were fitted with outcome as categorized levels (negative, acceptable/normal and above acceptable) of beta-lactam and tetracycline. The proportional odds assumption was valid for both beta-lactam (p = 0.3158) and tetracycline (p = 0.1866) residue levels when the Proportional Odds Model was fitted. Adjusting for beef sample characteristics, female cattle carcasses beef samples were four times more likely to have above acceptable beta-lactam residue levels compared to male cattle carcasses (OR = 4.16; 95% CL [1.35–12.80]; p = 0.131). In addition, beef samples of cattle carcasses whose place of origin was urban were less likely to have above acceptable beta-lactam residue levels compared to when cattle carcasses’ place of origin was rural (OR = 0.001; 95% CL [<0.01–0.01]; p<0.0001; Table 5). Similarly, beef samples of cattle carcasses whose place of origin was urban were less likely to have above acceptable tetracycline residues levels compared to when cattle carcasses’ place of origin was rural (OR = 0.17; 95% CL [0.05–0.62]; p = 0.0078; Table 5).

**Table 5:** Estimates obtained from proportional odds model.

Cattle Carcass Characteristics	P-value
**Beta-lactam**	
Sex Females	0.0131*
Age Adults	0.2385
Breed Cross	0.2645
Place Urban	< .0001*
**Tetracycline**	
Sex Females	0.1787
Age Adults	0.8007
Breed Cross	0.7995
Place Urban	0.0078*

The asterisk (*) denotes p<0.05. Female beef samples showed lower β-lactam residual levels than male (*p<0.05); for both β-lactam and tetracycline, urban cattle samples were less likely to contain above acceptable antimicrobial levels (*p<0.05) when the Proportional Odds Model was fitted.

Place of origin and sex of beef carcasses were independent predictors for above acceptable level of beta-lactam residues (p<0.05) while only place of origin of beef carcasses was an independent predictor for above acceptable level of and tetracycline antimicrobial residues. Breed and age of beef carcasses were not independent predictors for above acceptable level for both beta-lactam and tetracycline residues levels (p>0.05; Table 5). Further analysis entailed fitting two separate models with both beta-lactam and tetracycline residue levels as continuous outcomes. The SAS procedure Mixed (Proc Mixed) was used assuming an unstructured covariance structure. Beta-lactam residues levels as the outcome had a good fit Likelihood Ratio Statistic (LRT) p value<0.0001 implying sex, breed and age explained the variation observed in the beta-lactam residual levels. Adjusting for beef sample characteristics, age (p value = 0.019) and sex (p value = 0.011) were associated with the variations observed in the beta-lactam residual levels. The estimate -4.8 in female beef samples implied the beta-lactam residual levels were higher in male beef samples. In addition, the estimate for the adult beef samples 4.7 implied lower beta-lactam residual levels were associated with young beef samples. The estimate for local breed was -1.1 implying higher beta-lactam residual levels among cross breed samples although the difference was not statistically significant p = 0.578. On the other hand, tetracycline residue levels as outcome showed a poor fit LRT, p value = 0.5832 implying the model with covariates sex, breed, and age, was not plausible, and could not explain the variation observed in the tetracycline residual levels.

## Discussion

The goal of this study was to describe the occurrence of beta-lactam and tetracycline residues, two commonly used antimicrobials, in beef at abattoirs originating from rural and urban places in Uganda. In our study, antibiotic residues were generally below the recommended acceptable limits (designated as negative) for both tetracycline and β-lactam in the rural beef whereas those of urban beef were above the acceptable residue limits (designated as positive). Of the sampled beef of urban origin, 92.5% had antibiotic residues of which all contained β-lactam residues and 24.5% contained tetracycline residues. Conversely, of the sampled beef of rural origin 7.4% had antibiotic residues at a ratio of 1:2 of β-lactam to tetracycline antibiotic residues. Our findings however showed that beef samples of cattle carcasses whose place of origin was urban were less likely to have above acceptable beta-lactam residue levels compared to when cattle carcasses’ place of origin was rural. Likewise, beef samples of cattle carcasses whose place of origin was urban were less likely to have above acceptable tetracycline residues levels compared to when cattle carcasses’ place of origin was rural. There was thus a significant association between the origin of beef, whether rural or urban, and the presence of antibiotic residue in the samples analyzed. These findings imply that there is more antibiotic residue occurrence in the urban beef compared to the rural beef. While in the urban beef there was more β-lactam than tetracycline residues above the acceptable limits, there was more tetracycline than β-lactam antibiotic residues above the acceptable limits in the rural beef.

The findings of our research are supported by Olatoye and Ogandipe [33], indicating low antibiotic residues in rural compared to urban carcasses. Findings from the rural samples in our study were also comparable to those by Samandoulougou [13] and Mayuni [34], indicating tetracycline as the antibiotic with the highest antibiotic residue occurrence, while the urban results showed that β-lactams were the highest antibiotic residue occurring in cattle. Concurrent multiple drug usage of the β-lactams and tetracyclines is a pattern that may be more prominent in urban than in rural areas, leading to the presence of higher amounts of these residues in urban beef. The enterohepatic drug circulation for tetracyclines leads to drug persistence, thus the later use of β-lactams brings about multiple drug residue occurrence [35]. The disease status of the liver or kidney, for example in fasciolosis, significantly decreases tetracycline elimination half-life. Additionally, high-dose drug usage especially for the tetracyclines leads to prolonged residue occurrence even after the withdraw period.

Our results showed that the age of animals was significantly associated with antimicrobial residues in both rural and urban slaughters, although more significant in urban than rural samples. Urban slaughters tended more toward the adult cattle compared to young ones in the rural slaughters. In the rural slaughter places, there were equal proportions of young and adult slaughters. Although there were equal proportions of young to adult slaughters with β-lactam negative samples, there were more adult than young slaughters with tetracycline-negative samples. In contrast, for the β-lactam positive samples, the ratio of young to adult samples was 1:5 compared to 1:2 for tetracycline positive samples. This implies high antibiotic (β-lactam and tetracycline) residue occurrence in adult compared to young cattle, as previously established by Abdelgadir *et al* [36] in Khartoum. Data by Food Safety and Inspection Service USDA [29] showed contrasting results, suggesting that β-lactams are the antimicrobials with the highest drug residue in cattle compared to the tetracyclines, possibly due to the differences in the animal management systems and regulation enforcement. The unintended use of both drugs (β-lactam and tetracycline), especially in the fattening systems is mostly done in the semi-intensive and intensive farming systems involving indirect usage of antimicrobials (β-lactam) in feeds, drenches and drinking water, and using feed additives like vitamins, minerals and amino acids which may contain other antimicrobials (mostly tetracycline) leading to multiple drug residue occurrence. This could be the most probable cause since it is the commonest practice in peri-urban and urban farming.

Unlike the age of animals, sex of the animals was not a significant variable with respect to animals slaughtered at both rural and urban abattoirs. With almost similar male-to-male and female-to-female ratios in relation to urban to rural comparisons, together with the male-to-female ratio (rural sample) compared to male-to-female ratio (urban sample) comparison, the sex variable was not significant to both rural and urban slaughters.

As a variable, animal breed was significant with respect to both rural and urban slaughters, with urban slaughters tending more towards the local than crossbreeds, given the equal proportions of local and crossbreed slaughters in the rural abattoirs. Although there was equal proportion of local to crossbreed β-lactam negative samples, there were more local than crossbreed tetracycline negative samples. However, for the β-lactam positive samples, the ratio of local to crossbreeds was 4:1 compared to 2:1 in the tetracycline positive samples. This implies that there was high antibiotic residue occurrence in the locals than crosses, in contrast to the finding of Abdelgadir *et al* [36] whose study observed higher antibiotic residue in crossbreed cattle than in the local cattle. This was possibly due to the differences in breeds of interest, and cattle management systems, together with low sample size (n = 10) used, compared to other breeds’ sample sizes.

Our study findings are important for a number of reasons. First, data showing the use of antimicrobials in livestock are scarce in developing countries such as Uganda, even though such use has been shown by several recent studies to be increasing [37–39]. The lack of data is attributable to the lack of publicly funded surveillance systems, and the disinclination of food animal producers, feed producers, and veterinary pharmaceutical companies to provide comprehensive reports of antimicrobial consumption data used in livestock [38]. Our study thus provides a modest contribution to the data on the presence of antimicrobials in animal tissues consumed as food, and may be used as a baseline in future surveillance studies to monitor antimicrobial use in livestock in Uganda and the region. Second, a direct link has been made between presence of antimicrobials in animal tissues and antimicrobial drug resistance [27]. Landers et al. have extensively reviewed the evidence indicating an association between antibacterial drug use in food animals and the occurrence of antibiotic drug resistance in humans [28]. For low-income countries like Uganda that bear a high bacterial disease burden, widespread antimicrobial resistance acquired through consumption of beef and other animal products, or dissemination to the environment through animal use, may be highly consequential. Despite the substantial potential effect of the wanton consumption of antibiotics both in humans and animals, the use of antimicrobials in food animals such as cattle remains mostly unregulated [40]. Given the critical role of antimicrobials such as β-lactams and tetracyclines in limiting morbidity and mortality from a host of several bacterial infections, it is imperative that the widespread use of antimicrobials be curbed through regulatory provisions, policy, and enforcement to avert the potential crisis from inappropriate usage. Third, because of the projected increase in populations in low- and middle-income countries such as Uganda, industrial food animal production involving intensive practices geared at obtaining greater economies of scale and efficiency, are likely to increase in the coming decades [38,41]. These intensive food animal production methods will involve using subtherapeutic dosages of antimicrobials for growth promotion and disease prevention to meet the demands for meat in the rapidly growing economies and populations. It has been estimated that globally, antibiotic consumption in livestock will increase by 67% by 2030, and by 99% among rapidly industrializing countries such as Brazil, Russia, India, China, and South Africa [38].

A number of limitations were present in this study. First, this study did not consider other possible confounding factors including physiological conditions in the cattle such as liver and kidney function; feeding and cattle management systems; and body weight and body score on a comparison basis when case pairing. Second, because of inadequate record keeping by the cattle sellers and abattoir operators, and because of time constraints, the time lag between the use of the antibiotic and sampling time in comparison to the rural and urban beef case pairing was not considered. We also did not conduct a retrospective follow-up of cattle records to verify the origin of the cattle slaughtered in the abattoirs. Additionally, the study was unable to identify and quantify the specific β-lactam antimicrobial and other commonly used antimicrobials including streptomycin, gentamycin and erythromycin. Comparisons of case-pairs and seasonal variation of drug residues across all rural and urban beef carcasses were not feasible in this study. Regardless, this study showed that there is a significant difference in drug residue proportion of commonly used antimicrobials in Uganda’s rural and urban beef. Further, this study showed a significant difference in the drug residue proportion of the commonly used antimicrobials related to the age and breed of Uganda’s rural and urban cattle, although sex had no significant effect on the result.

In light of the findings from the present research, we recommend routine pre- and post-slaughter drug residue screening of all the livestock and carcasses in all the livestock slaughter places. The World Health Organization recently recommended discontinuation of the non-essential use of antimicrobials in livestock to promote growth or prevent disease, as part of the global action plan on antimicrobial resistance [37,42]. A recent global study has also shown that antibiotic consumption for approximately 16 years up to 2015, in low- and middle-income countries has been approaching, and in some countries, exceeding levels that are typically seen in high-income countries [39]. Screening for antimicrobial residues should be done in conjunction with the national drug residue monitoring and surveillance programs. Further research is warranted to determine the extent and burden of antibiotic resistance in humans in relation to drug residues in livestock in Uganda. Findings from such studies should guide the refinement of regulations and standards towards drug residue management for all stakeholders in the livestock industry in Uganda. These standards will include policies targeting the reduction of unnecessary antibiotic use in livestock, increased research on alternative antimicrobials such as plant-derived antimicrobials and probiotics, together with vaccination against some bacterial diseases that may be of great importance in the near future, and setting standardized national drug residue limits and acceptable daily intake values.

In conclusion, a significant proportion of antibiotic residues above the recommended residue limits occured in beef, differing across the place of origin (urban versus rural), age and breed, but not the sex of the cattle slaughtered in eight representative slaughterhouses in Uganda. These antibiotic residues present a big public health concern. Human consumption of such beef may be associated with the development of antibiotic resistant human pathogens and other potential health hazards that may present treatment challenges, and imperil human and food animal wellbeing.

## Supporting information

S1 Table This is the dataset for the tetracycline and beta-lactam in beef. (PDF)

S2 Table These are the summaries for the analyzed data. (PDF)

S3 Table These are the tables for fixed and random effects. (PDF)
