# Supplementary material for: Inappropriate usage of selected antimicrobials: Comparative residue proportions in rural and urban beef in Uganda
Source: PLoS One. 2019 Jan 10;14(1):e0209006. doi: 10.1371/journal.pone.0209006 (PMC6328120; doi:10.1371/journal.pone.0209006)
Supplement: S1 Table — (PDF) [file pone.0209006.s001.pdf]

#	Tetracycline Tet-µg/kg	Beta-lactam Beta-µg/kg	Sex	Age	Breed	Origin	
1	2.7	3.03	F	Y	L	R	Y=young
2	1.9	1.973	F	Y	X	R	A=adult
3	2.3	2.065	F	A	L	R	L=local
4	6.9	2.263	M	Y	L	R	X=crossbreed
5	0.9	2.231	M	Y	X	R	R=rural
6	1.2	3.073	F	A	X	R	U=urban
7	12.7	2.785	F	A	L	R	
8	2	3.848	M	A	X	R	
9	6.1	1.527	M	Y	L	R	
10	5.5	2.328	F	A	L	R	
11	2.7	2.037	F	A	L	R	
12	9.8	2.785	F	A	L	R	
13	4	4.352	F	A	X	R	
14	2.6	2.785	F	A	X	R	
15	9.6	1.109	F	A	X	R	
16	8.8	1.966	F	Y	X	R	
17	1	2.785	M	Y	L	R	
18	7.5	4.636	M	A	L	R	
19	5.334	3.426	F	A	X	R	
20	7.642	5.133	M	Y	X	R	
21	25.85	2.48	M	Y	L	R	
22	3.679	14.48	M	Y	X	R	
23	1.85	1.433	M	A	X	R	
24	4.351	2.022	F	A	L	R	
25	19.858	2.124	M	A	X	R	
26	8.179	16.318	M	Y	X	R	
27	3.985	3.105	M	Y	X	R	
28	6.861	2.794	M	Y	X	R	
29	2.333	4.507	F	A	X	R	
30	1.615	3.524	M	Y	X	R	
31	8.967	2.55	F	A	L	R	
32	13.86	2.824	F	Y	L	R	
33	2.527	2.915	M	Y	L	R	
34	4.7	3.539	F	A	L	R	
35	3	2.932	M	Y	X	R	
36	6.1	2.488	F	A	X	R	
37	4.1	2.685	M	Y	L	R	
38	3	3.015	M	Y	X	R	
39	5.1	2.409	M	A	L	R	
40	3.7	2.6	F	A	L	R	
41	6.1	2.128	M	Y	L	R	
42	7.6	2.02	M	A	X	R	
43	2.7	2.278	F	Y	X	R	
44	4	2.508	F	Y	X	R	

45	6.6	2.663	M	Y	L	R
46	6.1	2.991	M	A	L	R
47	3.2	1.857	M	Y	L	R
48	5.1	1.949	M	A	L	R
49	2.4	2.39	F	A	L	R
50	2.3	2.817	M	A	X	R
51	2.4	2.75	M	Y	X	R
52	2.5	2.548	M	Y	X	R
53	1.8	1.77	F	A	X	R
54	4.3	1.508	F	Y	L	R
55	2.6	2.145	F	Y	X	R
56	2.5	1.933	F	A	L	R
57	3.4	2.333	M	Y	X	R
58	4.7	2.069	M	Y	X	R
59	3	4.072	M	Y	X	R
60	2.4	3.346	F	A	L	R
61	6.2	2.75	M	Y	L	R
62	5.1	1.784	M	A	X	R
63	2.7	1.865	M	A	L	R
64	1.6	1.66	M	Y	X	R
65	9.4	0.827	F	Y	L	R
66	1.4	2.171	F	A	L	R
67	6.1	2.488	M	A	X	R
68	9.1	2.197	F	A	L	R
69	5.9	2.028	F	Y	L	R
70	8.1	2.012	F	A	L	R
71	4	2.004	F	A	L	R
72	5.8	2.094	F	A	L	R
73	5.3	3.254	M	Y	L	R
74	1.6	4.239	F	Y	L	R
75	6	2.874	F	A	L	R
76	1.6	2.794	F	Y	L	R
77	4	3.126	F	Y	L	R
78	3	4.519	F	A	L	R
79	2.3	2.39	F	A	L	R
80	2.4	2.206	F	A	L	R
81	5.2	3.28	F	A	L	R
82	0.3	8.3	F	Y	L	U
83	0.2	18	M	A	L	U
84	43.8	37.5	M	A	L	U
85	4.7	12.7	F	A	L	U
86	2.4	26	F	A	L	U
87	0.7	24.8	F	A	L	U
88	0.8	23.7	F	A	L	U
89	0.7	13.6	F	Y	X	U
90	0.4	11.1	F	A	X	U
91	0.5	9.2	F	Y	X	U

92	65	34.3	F	Y	L	U
93	1.8	19.3	F	Y	L	U
94	2.1	17.8	F	Y	X	U
95	0.7	27.8	M	A	L	U
96	5.2	11.9	F	A	X	U
97	4.7	24.3	M	A	X	U
98	3.1	7.6	F	Y	L	U
99	3.9	16.8	F	Y	L	U
100	25.9	31.4	M	Y	L	U
101	44.1	39.9	F	A	L	U
102	1.2	10.6	F	A	L	U
103	1.1	29.9	M	A	L	U
104	2.4	7.7	F	Y	L	U
105	3.9	33.3	F	Y	L	U
106	63.5	39.8	M	A	L	U
107	37.4	62.1	M	A	L	U
108	1.3	36.2	M	A	L	U
109	12.8	54.7	F	A	L	U
110	1.2	39.1	M	A	L	U
111	61.4	60.9	M	A	L	U
112	72.8	57.1	F	A	X	U
113	3.9	41.2	F	A	L	U
114	9.2	35.3	F	A	X	U
115	0.9	38.5	M	A	X	U
116	70.3	77	M	A	X	U
117	14	48	M	A	X	U
118	2.9	17.3	M	A	L	U
119	76.2	76.3	F	A	L	U
120	0.3	21.9	M	A	L	U
121	0.2	48.8	F	A	L	U
122	0.4	23.6	F	A	L	U
123	0.4	28.1	F	A	L	U
124	4.7	33.4	M	A	L	U
125	4.4	24.3	F	A	L	U
126	0.8	19.1	M	A	L	U
127	2.5	11.6	F	A	L	U
128	2.4	16.1	M	A	L	U
129	2.4	24.4	F	A	L	U
130	3	26.4	M	A	L	U
131	4.3	14.8	F	A	L	U
132	3.9	10.9	F	A	L	U
133	1.6	36.4	M	A	L	U
134	1.3	28.5	F	A	L	U
