# Supplementary material for: Inappropriate usage of selected antimicrobials: Comparative residue proportions in rural and urban beef in Uganda
Source: PLoS One. 2019 Jan 10;14(1):e0209006. doi: 10.1371/journal.pone.0209006 (PMC6328120; doi:10.1371/journal.pone.0209006)
Supplement: S2 Table — (PDF) [file pone.0209006.s002.pdf]

The Mixed Procedure

| Model Information | |
|---|---|
| Data Set | LIB.ANTIBIOTICS |
| Dependent Variable | Tetracycline |
| Covariance Structure | Unstructured |
| Subject Effect | place |
| Estimation Method | REML |
| Residual Variance Method | Profile |
| Fixed Effects SE Method | Model-Based |
| Degrees of Freedom Method | Containment |

| Class Level Information | | |
|---|---|---|
| Class | Levels | Values |
| place | 2 | Rural Urban |
| Sex | 2 | F M |
| Age | 2 | A Y |
| Breed | 2 | L X |

| Dimensions | |
|---|---|
| Covariance Parameters | 7 |
| Columns in X | 7 |
| Columns in Z per Subject | 3 |
| Subjects | 2 |
| Max Obs per Subject | 81 |

| Number of Observations | |
|---|---|
| Number of Observations Read | 134 |
| Number of Observations Used | 134 |
| Number of Observations Not Used | 0 |

| Iteration History | | | |
|---|---|---|---|
| Iteration | Evaluations | -2 Res Log Like | Criterion |
| 0 | 1 | 1085.362197 | |
| 1 | 1 | 1080.665136 | 0 |

Convergence criteria met but final Hessian is not positive definite.

| Covariance Parameter Estimates | | |
|---|---|---|
| Cov Parm | Subject | Estimate |
| UN(1,1) | place | 25.4657 |
| UN(2,1) | place | 0 |
| UN(2,2) | place | 0.000011 |
| UN(3,1) | place | 0 |
| UN(3,2) | place | 0 |
| UN(3,3) | place | 0.000011 |
| Residual | | 209 |

| Fit Statistics | | |
|---|---|---|
| -2 Res Log Likelihood | 1080.7 | |
| AIC (Smaller is Better) | | 1094.7 |
| AICC (Smaller is Better) | | 1095.6 |
| BIC (Smaller is Better) | | 1085.5 |

| Null Model Likelihood Ratio Test | | |
|---|---|---|
| DF | Chi-Square | Pr > ChiSq |
| 6 | 4.7 | 0.5832 |

**Solution for Fixed Effects**

| Effect | Sex | Age | Breed | Estimate | Standard Error | DF | t Value | Pr > \|t\| |
|---|---|---|---|---|---|---|---|---|
| Intercept | | | | 9.2649 | 4.5673 | 0 | 2.03 | . |
| Sex | F | | | -2.6916 | 2.5786 | 129 | -1.04 | 0.2985 |
| Sex | M | | | 0 | . | . | . | . |
| Age | | A | | 1.8589 | 2.7219 | 129 | 0.68 | 0.4959 |
| Age | | Y | | 0 | . | . | . | . |
| Breed | | | L | -0.1712 | 2.7554 | 129 | -0.06 | 0.9506 |
| Breed | | | X | 0 | . | . | . | . |

**Solution for Random Effects**

| Effect | place | Estimate | Std Err Pred | DF | t Value | Pr > \|t\| |
|---|---|---|---|---|---|---|
| Intercept | Rural | -3.3363 | 3.7861 | 129 | -0.88 | 0.3798 |
| place | Rural | -1.44E-06 | 0.003313 | 129 | 0 | 0.9997 |
| place | Rural | 0 | 0.003313 | 129 | 0 | 1 |
| Intercept | Urban | 3.3363 | 3.7861 | 129 | 0.88 | 0.3798 |
| place | Urban | 0 | 0.003313 | 129 | 0 | 1 |
| place | Urban | 1.44E-06 | 0.003313 | 129 | 0 | 0.9997 |

**Type 3 Tests of Fixed Effects**

| Effect | Num DF | Den DF | F Value | Pr > F |
|---|---|---|---|---|
| Sex | 1 | 129 | 1.09 | 0.2985 |
| Age | 1 | 129 | 0.47 | 0.4959 |
| Breed | 1 | 129 | 0 | 0.9506 |



The Mixed Procedure

**Model Information**

| | |
|---|---|
| Data Set | LIB.ANTIBIOTICS |
| Dependent Variable | Betalactam |
| Covariance Structure | Unstructured |
| Subject Effect | place |
| Estimation Method | REML |
| Residual Variance Method | Profile |
| Fixed Effects SE Method | Model-Based |
| Degrees of Freedom Method | Containment |

**Class Level Information**

| Class | Levels | Values |
|---|---|---|
| place | 2 | Rural Urban |
| Sex | 2 | F M |
| Age | 2 | A Y |
| Breed | 2 | L X |

**Dimensions**

| | |
|---|---|
| Covariance Parameters | 7 |
| Columns in X | 7 |
| Columns in Z per Subject | 3 |
| Subjects | 2 |
| Max Obs per Subject | 81 |

**Number of Observations**

| | |
|---|---|
| Number of Observations Read | 134 |
| Number of Observations Used | 134 |

| | |
|---|---|
| **Number of Observations Not Used** | 0 |

**Iteration History**

| Iteration | Evaluations | -2 Res Log Like | Criterion |
|---|---|---|---|
| 0 | 1 | 1102.751728 | |
| 1 | 1 | 998.387682 | 0 |

Convergence criteria met but final Hessian is not positive definite.

**Covariance Parameter Estimates**

| Cov Parm | Subject | Estimate |
|---|---|---|
| UN(1,1) | place | 326.61 |
| UN(2,1) | place | 0 |
| UN(2,2) | place | 5.69E-06 |
| UN(3,1) | place | 0 |
| UN(3,2) | place | 0 |
| UN(3,3) | place | 5.69E-06 |
| Residual | | 108.39 |

**Fit Statistics**

| | |
|---|---|
| -2 Res Log Likelihood | 998.4 |
| AIC (Smaller is Better) | 1012.4 |
| AICC (Smaller is Better) | 1013.3 |
| BIC (Smaller is Better) | 1003.2 |

**Null Model Likelihood Ratio Test**

| DF | Chi-Square | Pr > ChiSq |
|---|---|---|
| 6 | 104.36 | <.0001 |

**Solution for Fixed Effects**

| Effect | Sex | Age | Breed | Estimate | Standard Error | DF | t Value | Pr > \|t\| |
|---|---|---|---|---|---|---|---|---|
| Intercept | | | | 16.4637 | 12.9455 | 0 | 1.27 | . |
| Sex | F | | | -4.7682 | 1.857 | 129 | -2.57 | 0.0114 |
| Sex | M | | | 0 | . | . | . | . |
| Age | | A | | 4.6739 | 1.9671 | 129 | 2.38 | 0.019 |
| Age | | Y | | 0 | . | . | . | . |
| Breed | | | L | -1.1083 | 1.9885 | 129 | -0.56 | 0.5782 |
| Breed | | | X | 0 | . | . | . | . |

**Solution for Random Effects**

| Effect | place | Estimate | Std Err Pred | DF | t Value | Pr > \|t\| |
|---|---|---|---|---|---|---|
| Intercept | Rural | -12.742 | 12.8159 | 129 | -0.99 | 0.322 |
| place | Rural | -2.22E-07 | 0.002386 | 129 | 0 | 0.9999 |
| place | Rural | 0 | 0.002386 | 129 | 0 | 1 |
| Intercept | Urban | 12.742 | 12.8159 | 129 | 0.99 | 0.322 |
| place | Urban | 0 | 0.002386 | 129 | 0 | 1 |
| place | Urban | 2.22E-07 | 0.002386 | 129 | 0 | 0.9999 |

**Type 3 Tests of Fixed Effects**

| Effect | Num DF | Den DF | F Value | Pr > F |
|---|---|---|---|---|
| Sex | 1 | 129 | 6.59 | 0.0114 |
| Age | 1 | 129 | 5.65 | 0.019 |
| Breed | 1 | 129 | 0.31 | 0.5782 |

The Mixed Procedure

| Model Information | |
|---|---|
| **Data Set** | LIB.ANTIBIOTICS |
| **Dependent Variable** | Betalactam |
| **Covariance Structure** | Unstructured |
| **Subject Effect** | place |
| **Estimation Method** | REML |
| **Residual Variance Method** | Profile |
| **Fixed Effects SE Method** | Model-Based |
| **Degrees of Freedom Method** | Containment |

| Class Level Information | | |
|---|---|---|
| **Class** | **Levels** | **Values** |
| **place** | 2 | Rural Urban |
| **Sex** | 2 | F M |
| **Age** | 2 | A Y |
| **Breed** | 2 | L X |

| Dimensions | |
|---|---|
| **Covariance Parameters** | 7 |
| **Columns in X** | 5 |
| **Columns in Z per Subject** | 3 |
| **Subjects** | 2 |
| **Max Obs per Subject** | 81 |

| Number of Observations | |
|---|---|
| **Number of Observations Read** | 134 |
| **Number of Observations Used** | 134 |
| **Number of Observations Not Used** | 0 |

| Iteration History | | | |
|---|---|---|---|
| **Iteration** | **Evaluations** | **-2 Res Log Like** | **Criterion** |
| **0** | 1 | 1108.360575 | |
| **1** | 1 | 1001.909066 | 0 |

Convergence criteria met but final Hessian is not positive definite.

| Covariance Parameter Estimates | | |
|---|---|---|
| **Cov Parm** | **Subject** | **Estimate** |
| **UN(1,1)** | **place** | 321.36 |
| **UN(2,1)** | **place** | 0 |
| **UN(2,2)** | **place** | 5.66E-06 |
| **UN(3,1)** | **place** | 0 |
| **UN(3,2)** | **place** | 0 |
| **UN(3,3)** | **place** | 5.66E-06 |
| **Residual** | | 107.83 |

| Fit Statistics | |
|---|---|
| **-2 Res Log Likelihood** | 1001.9 |
| **AIC (Smaller is Better)** | 1015.9 |
| **AICC (Smaller is Better)** | 1016.8 |
| **BIC (Smaller is Better)** | 1006.8 |

| Null Model Likelihood Ratio Test | | |
|---|---|---|
| **DF** | **Chi-Square** | **Pr > ChiSq** |
| 6 | 106.45 | <.0001 |

**Solution for Fixed Effects**

| Effect | Sex | Age | Estimate | Standard Error | DF | t Value | Pr > \|t\| |
|---|---|---|---|---|---|---|---|
| Intercept | | | 15.8817 | 12.8007 | 0 | 1.24 | . |
| Sex | F | | -4.928 | 1.83 | 130 | -2.69 | 0.008 |
| Sex | M | | 0 | . | . | . | . |
| Age | | A | 4.5499 | 1.9494 | 130 | 2.33 | 0.0211 |
| Age | | Y | 0 | . | . | . | . |

**Solution for Random Effects**

| Effect | place | Estimate | Std Err Pred | DF | t Value | Pr > \|t\| |
|---|---|---|---|---|---|---|
| Intercept | Rural | -12.6401 | 12.7117 | 130 | -0.99 | 0.3219 |
| place | Rural | -2.23E-07 | 0.002379 | 130 | 0 | 0.9999 |
| place | Rural | 0 | 0.002379 | 130 | 0 | 1 |
| Intercept | Urban | 12.6401 | 12.7117 | 130 | 0.99 | 0.3219 |
| place | Urban | 0 | 0.002379 | 130 | 0 | 1 |
| place | Urban | 2.23E-07 | 0.002379 | 130 | 0 | 0.9999 |

**Type 3 Tests of Fixed Effects**

| Effect | Num DF | Den DF | F Value | Pr > F |
|---|---|---|---|---|
| Sex | 1 | 130 | 7.25 | 0.008 |
| Age | 1 | 130 | 5.45 | 0.0211 |
