# Supplementary material for: Inappropriate usage of selected antimicrobials: Comparative residue proportions in rural and urban beef in Uganda
Source: PLoS One. 2019 Jan 10;14(1):e0209006. doi: 10.1371/journal.pone.0209006 (PMC6328120; doi:10.1371/journal.pone.0209006)
Supplement: S3 Table — (PDF) [file pone.0209006.s003.pdf]

Solution for Fixed Effects			
Effect	Estimate	Standard Error	P Value
Intercept	16.5	12.9	
Sex Females	-4.8	1.9	0.011
Sex Males			
Age Adults	4.7	2.0	0.019
Age Young			
Breed Local	-1.1	2.0	0.578
Breed Cross			

. .

Solution for Random Effects				
Effect	place	Estimate	Std Err Pred	P Value
Intercept	Rural	-12.7	12.816	0.322
place	Rural	0.0	0.002	1.000
place	Rural	0.0	0.002	1.000
Intercept	Urban	12.7	12.816	0.322
place	Urban	0.0	0.002	1.000
place	Urban	0.0	0.002	1.000

Type 3 Tests of Fixed Effects		
Effect	F Value	P Value
Sex	6.59	0.011
Age	5.65	0.019
Breed	0.31	0.578

Solution for Fixed Effects			
Effect	Estimate	Standard Error	P Value
Intercept	9.3	4.6	
Sex Females	-2.7	2.6	0.299
Sex Males	0.0		
Age Adults	1.9	2.7	0.496
Age Young	0.0		
Breed Local	-0.2	2.8	0.951
Breed Cross	0.0		

. .

Solution for Random Effects				
Effect	place	Estimate	Std Err Pred	P Value
Intercept	Rural	-3.3	3.786	0.380
place	Rural	0.0	0.003	1.000
place	Rural	0.0	0.003	1.000
Intercept	Urban	3.3	3.786	0.380
place	Urban	0.0	0.003	1.000
place	Urban	0.0	0.003	1.000

Type 3 Tests of Fixed Effects		
Effect	F Value	P Value
Sex	1.09	0.299
Age	0.47	0.496
Breed	0	0.951
